# Improvement in coronary haemodynamics after percutaneous coronary intervention: assessment using instantaneous wave-free ratio

**DOI:** 10.1136/heartjnl-2013-304387

**Published:** 2013-09-18

**Authors:** Sukhjinder S Nijjer, Sayan Sen, Ricardo Petraco, Rajesh Sachdeva, Florim Cuculi, Javier Escaned, Christopher Broyd, Nicolas Foin, Nearchos Hadjiloizou, Rodney A Foale, Iqbal Malik, Ghada W Mikhail, Amarjit S Sethi, Mahmud Al-Bustami, Raffi R Kaprielian, Masood A Khan, Christopher S Baker, Michael F Bellamy, Alun D Hughes, Jamil Mayet, Rajesh K Kharbanda, Carlo Di Mario, Justin E Davies

**Affiliations:** 1 Imperial College London, London, UK; 2 Wellstar Cardiology, North Fulton Hospital, Roswell, Georgia, USA; 3 VAMC, Little Rock, Arkansas, USA; 4 John Radcliffe Hospital, Oxford, UK; 5 Cardiovascular Institute, Hospital Clínico San Carlos, Madrid, Spain; 6 NIHR Cardiovascular Biomedical Research Unit, Royal Brompton Hospital, Imperial College, London, UK

**Keywords:** CORONARY PHYSIOLOGY

## Abstract

**Objective:**

To determine whether the instantaneous wave-free ratio (iFR) can detect improvement in stenosis significance after percutaneous coronary intervention (PCI) and compare this with fractional flow reserve (FFR) and whole cycle Pd/Pa.

**Design:**

A prospective observational study was undertaken in elective patients scheduled for PCI with FFR ≤0.80. Intracoronary pressures were measured at rest and during adenosine-mediated vasodilatation, before and after PCI. iFR, Pd/Pa and FFR values were calculated using the validated fully automated algorithms.

**Setting:**

Coronary catheter laboratories in two UK centres and one in the USA.

**Patients:**

120 coronary stenoses in 112 patients were assessed. The mean age was 63±10 years, while 84% were male; 39% smokers; 33% with diabetes. Mean diameter stenosis was 68±16% by quantitative coronary angiography.

**Results:**

Pre-PCI, mean FFR was 0.66±0.14, mean iFR was 0.75±0.21 and mean Pd/Pa 0.83±0.16. PCI increased all indices significantly (FFR 0.89±0.07, p<0.001; iFR 0.94±0.05, p<0.001; Pd/Pa 0.96±0.04, p<0.001). The change in iFR after intervention (0.20±0.21) was similar to ΔFFR 0.22±0.15 (p=0.25). ΔFFR and ΔiFR were significantly larger than resting ΔPd/Pa (0.13±0.16, both p<0.001). Similar incremental changes occurred in patients with a higher prevalence of risk factors for microcirculatory disease such as diabetes and hypertension.

**Conclusions:**

iFR and FFR detect the changes in coronary haemodynamics elicited by PCI. FFR and iFR have a significantly larger dynamic range than resting Pd/Pa. iFR might be used to objectively document improvement in coronary haemodynamics following PCI in a similar manner to FFR.

## Introduction

The instantaneous wave-free ratio (iFR) is an invasive, pressure-only index of coronary stenosis severity measured without pharmacological vasodilatation. It is calculated over five heart beats as the ratio of distal to proximal coronary pressures during the diastolic wave-free period of the cardiac cycle, when distal intracoronary resistance is stable and minimal.^1^ In ADVISE and ADVISE-Registry studies, and in an independent blinded cohort (the South Korean prospective study), the stenosis severity classification of iFR matched fractional flow reserve (FFR) in over 80% cases^1–3^; this was higher when accounting for the test-retest variability of FFR around its threshold.^2^ CLARIFY further demonstrated high diagnostic agreement with hyperaemic stenosis resistance a flow-based index of ischaemia.^4^

However, it is unknown whether iFR can detect changes in coronary haemodynamics immediately following percutaneous coronary intervention (PCI). If detectable, they may be useful to document improvements in haemodynamics following PCI.

In this study, we explored whether (1) iFR changed in patients undergoing PCI and (2) whether the size of the increment was similar in proportion to measurements obtained under adenosine-mediated hyperaemic conditions.

## Methods

### Study population

Patients with angina undergoing elective coronary angioplasty for clinical reasons were prospectively enrolled for pressure wire assessment before and after intervention. FFR was used as the reference standard to detect significant epicardial stenoses and only patients with physiologically significant lesions (FFR values ≤0.80) were included.

Diabetes was defined as the use of oral hypoglycaemic agents or subcutaneous insulin injection. Hypertension was defined by a formal diagnosis by the referring physician. Hypercholesterolaemia was defined as requiring a statin with total cholesterol ≥5 mmol/L and low density lipoprotein (LDL)≥2 mmol/L. Smoking status was patient reported and dichotomised as currently smoking cigarettes (including recent discontinuation within 1 year) and those who are never smokers or stopped over 1 year ago.

Patients were recruited from the Imperial College Healthcare NHS Trust, the Veterans Affairs Medical Center, Little Rock, Arkansas, USA and the John Radcliffe Hospital, Oxford, UK. The protocol was approved by local institutional review boards and ethics committees and patients provided written informed consent (NRES 09/H0712/102; NCT01118481).

### Study protocol: coronary catheterisation

Coronary angiography and pressure wire assessments of coronary stenoses were performed using conventional approaches. Intracoronary nitrates were administered in all cases before pressure wires were introduced. Pressure wires were normalised at the coronary ostia before every pressure recording. If more than one stent was used within one coronary segment, the pressure analysis was performed for the complete segment. For postangioplasty measurements, all stents were optimised with postdilation where angiographically indicated before further assessment with the pressure wire. Repeated measurements were performed after the angioplasty balloon had been removed, the catheter flushed and nitrates administered again. The pressure wire was normalised at the vessel ostium and then measurements were made at the same coronary location as pre-angioplasty.

All patients received an oral loading dose of aspirin 300 mg and clopidogrel 600 mg, and intravenous heparin according to weight, together with bivalirudin or GPII_b_III_a_-antagonist according to clinical indication.

### Haemodynamic recordings

Pressure wire recordings were made using the Pressure Wire Aeris (St. Jude Medical, Minneapolis, Minnesota) and Prestige pressure guide wire (Volcano Corporation, San Diego, California). Digital haemodynamic data were extracted from data storage systems (Radiview, St Jude Medical and ComboMap, Volcano Corporation) and processed off-line in a core laboratory using a custom software package with Matlab (Mathworks, Inc., Natick, Massachusetts).

### Calculation of Pd/Pa, iFR and FFR

**Figure 1:**
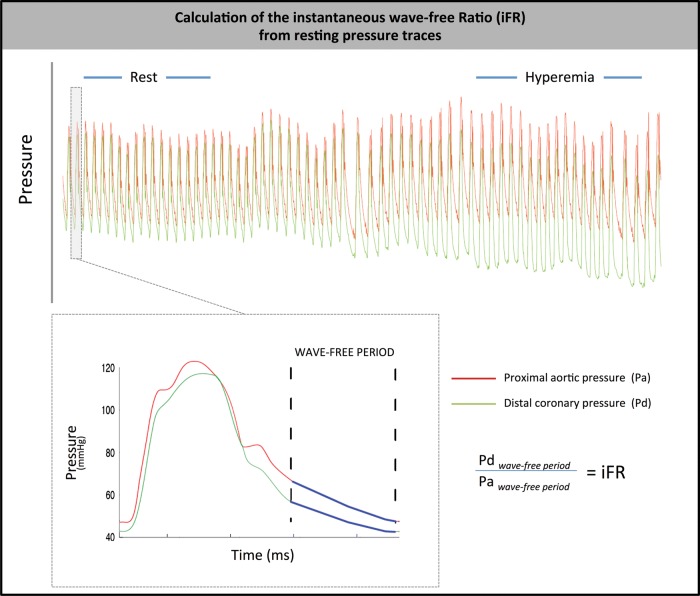
Calculation of iFR over the resting wave-free window. Using an automated off-line algorithm, iFR was calculated at rest from the distal-to-proximal pressure ratio during the wave-free period.

iFR was calculated as a ratio of the distal coronary pressure to proximal coronary pressure at rest, using the validated automated algorithms with phase alignment acting over the diastolic wave-free period over a minimum of five beats. iFR is measured using pressure-only, at baseline, without adenosine administration^1^(figure 1).

Pd/Pa ratio was calculated using the ratio of distal coronary pressure to proximal coronary pressure at rest over the entire cardiac cycle.

FFR measurements were performed using a standard technique,^5^ using the ratio of distal coronary pressure to proximal pressure during conditions of stable hyperaemia. Hyperaemia was induced by adenosine infusion at a rate of 140 mcg/kg/min, administered by femoral venous access in 96 (80%) stenoses and an intracoronary 60 mcg bolus in 24 (20%) stenoses.

### Data analysis

Statistical analysis was performed using Matlab (Mathworks Inc, Massachusetts, USA) and STATA V.11 (StataCorp, Texas). Values are expressed as mean±SD. Continuous variables were compared using the Student t test or Mann-Whitney U test. Subgroup data was assessed using ANOVA with repeated measures and the Bonferroni correction for multiple testing errors. The relationship between the change in pressure wire indices and stenosis severity based upon quantitative coronary angiography (QCA) were quantified using Pearson's product moment correlation coefficient. This study had 90% power to detect a difference of a 0.03 or greater difference between the delta in iFR and FFR after PCI. A p value <0.05 was considered statistically significant.

## Results

### Patient characteristics

A hundred and twelve patients (63±10 years old, 84% male) with 120 coronary stenoses were included. Patient demographics are shown in table 1. QCA demonstrated a mean diameter stenosis of 68±16%, and lesion length of 15.6±9.2 mm.

**Table 1:** Patient demographic data

	Number (%)
*Patients*	112
Age, yrs	63±10
Male	94 (84)
Diabetes	37 (33)
Smoker	44 (39)
Hypertension	90 (80)
Hyperlipidaemia	92 (82)
Renal failure on dialysis	3 (3)
Previous myocardial infarction	18 (16)
Impaired LV function EF<30%	13 (12)
Previous CABG	14 (13)
Stable angina	91 (81)
Unstable angina	21 (19)
Single-vessel disease	51 (46)
Multivessel disease	61 (54)
*Stenoses*	120
Coronary vessel	
Left main stem	2 (2)
Left anterior descending	63 (53)
Diagonal	4 (3)
Intermediate	2 (2)
Circumflex	15 (13)
Obtuse marginal	6 (5)
Right coronary	21 (18)
Posterior descending	1 (1)
Saphenous vein graft	6 (5)
Lesion location in vessel	
Proximal	62 (52)
Mid	52 (43)
Distal	6 (5)
Lesion characteristics	
Lesion severity (QCA %)	68±16
Lesion length (QCA mm)	15.6±9.2
Adenosine administration	
Central intravenous	96 (80)
Intracoronary bolus	24 (20)

Values are n, mean±SD or n (%). Risk factors are defined in the text.

CABG, coronary artery bypass grafting; EF, ejection fraction; LV, left ventricular; QCA, quantitative coronary angiography.

### Haemodynamic parameters

The mean haemodynamic parameters at rest and during adenosine infusion, before and after PCI are shown in table 2. Adenosine significantly increased heart rate and reduced systolic, diastolic and mean blood pressures compared with resting values and this occurred before and after angioplasty. The delta (Δ) in each parameter at rest, before and after PCI, was not significantly different from the hyperaemic delta before and after PCI (p>0.12). Responses did not differ within subgroups conventionally associated with higher levels of microcirculatory disease including people with diabetes, hypertension or current smokers (table 2).

**Table 2:** Haemodynamic changes observed at rest and during adenosine-mediated hyperaemia, before and after coronary angioplasty

	Preangioplasty			Postangioplasty			Delta		
Haemodynamic parameter	Resting	Adenosine	Rest versus adenosine p value	Resting	Adenosine	Rest versus adenosine p value	Resting pre–post	Adenosine pre-post	Rest versus adenosine p value
All stenoses									
Heart rate	68±12	73±12	<0.001	68±13	73±14	<0.001	0±7	1±7	0.21
Mean systolic pressure	119±27	104±26	<0.001	124±27	105±28	<0.001	5±25	1±24	0.12
Mean diastolic pressure	66±14	56±14	<0.001	69±13	58±14	<0.001	3±12	0±13	0.15
Mean arterial pressure	80±16	68±16	<0.001	84±16	69±18	<0.001	3±15	1±16	0.14
Diabetes									
Heart rate	70±12	72±12	0.01	69±13	73±12	0.003	0±4	1±5	0.56
Systolic blood pressure	121±26	111±14	0.002	122±31	106±32	<0.001	2±23	−4±19	0.17
Diastolic blood pressure	65±14	59±13	0.001	66±14	56±16	<0.001	1±10	−3±11	0.14
Mean arterial pressure	80±17	71±14	<0.001	81±17	68±20	<0.001	1±13	−3±14	0.15
Hypertension									
Heart rate	66±13	68±12	0.03	66±13	70±13	<0.001	0±4	2±6	0.41
Systolic blood pressure	109±25	101±25	0.01	113±26	101±23	<0.001	5±22	0±25	0.2
Diastolic blood pressure	61±13	56±13	0.01	64±14	56±12	<0.001	3±11	0±14	0.23
Mean arterial pressure	74±16	67±15	0.002	78±16	67±15	<0.001	3±13	1±17	0.23
Smoking									
Heart rate	70±13	76±13	<0.001	70±14	75±15	<0.001	0±9	−1±9	0.98
Systolic blood pressure	127±27	110±26	<0.001	128±31	111±32	<0.001	2±28	−2±25	0.33
Diastolic blood pressure	68±14	58±14	<0.001	70±14	59±14	<0.001	1±14	−2±13	0.33
Mean arterial pressure	84±17	72±15	<0.001	86±18	72±18	<0.001	2±16	−2±16	0.27

### Preangioplasty stenosis evaluation

Mean FFR was 0.66±0.14 (median 0.72, 0.56–0.78); mean Pd/Pa was 0.83±0.16 (median 0.90, 0.81–0.93); mean iFR was 0.75±0.21 (median 0.84, 0.65–0.89). The overall mean FFR was similar to the FAME and FAME-II studies.^6^ ^7^ Eighty-four stenoses (70%) were within FFR 0.6–0.80 range (with mean FFR 0.75±0.05, mean iFR 0.85±0.08, mean Pd/Pa 0.91±0.04) while 71 stenoses (59%) were within FFR 0.70–0.80 range (mean FFR 0.76±0.03, mean iFR 0.86±0.07, mean Pd/Pa 0.91±0.04). Using the iFR 0.90 cut-off to correspond to the FFR 0.80 cut-off, 86% classification match was found in this cohort.

Intracoronary adenosine was used in 24 stenoses: there was no significant difference in the FFR values measured using intracoronary adenosine versus intravenous adenosine infusion, either before (p=0.83) and or after PCI (p=0.79).

### Resting indices can be lower than hyperaemic indices

**Figure 2:**
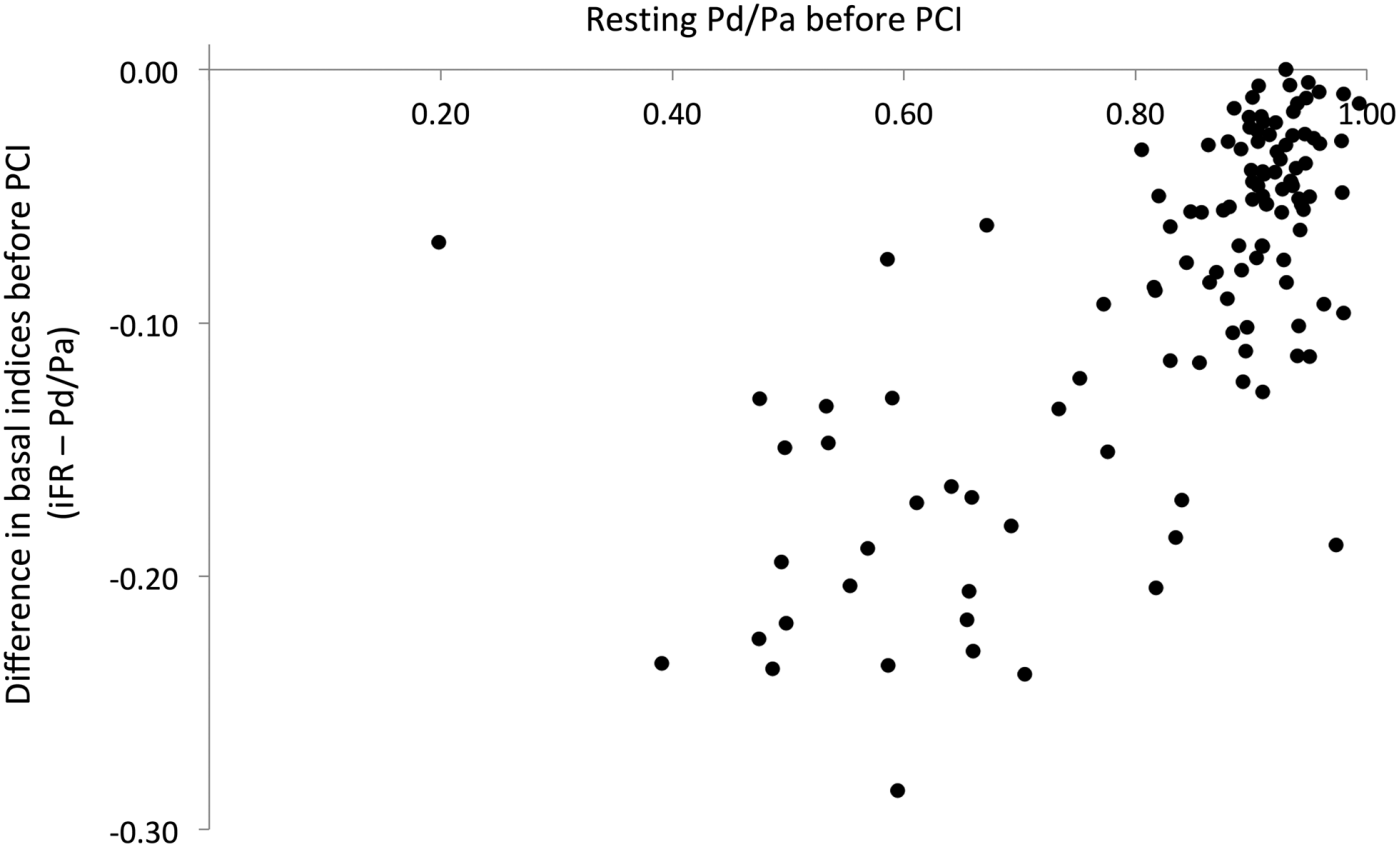
The difference in preintervention iFR and Pd/Pa values. The difference between iFR and Pd/Pa is shown against preintervention Pd/Pa values. iFR was lower than Pd/Pa by a mean of 0.08±0.07 across all stenoses.

In 26 stenoses (22%) the pre-PCI iFR value was numerically lower than FFR (iFR 0.45±0.20, FFR 0.55±0.16). This is a similar proportion to that reported in other studies.^1^ ^2^ ^4^ ^8^ ^9^ These lesions were anatomically more severe (diameter stenosis: 75±14% vs 66±16%, p=0.01) and were physiologically more significant (FFR 0.55±0.16 vs 0.69±0.12, p<0.01) than the remainder of the study population. There was no significant difference in heart rate or systolic and diastolic pressures between these individuals and the others (p≥0.10). There was also no significant difference in the rates of diabetes mellitus, hypertension or smoking status (p≥0.26); although hyperlipidaemia was more common in the group where FFR was lower than iFR (75% vs 87%, p=0.03). In contrast, Pd/Pa was numerically lower than FFR in only three stenoses (2.5%; values 0.70, 0.20, 0.50) and in all three cases iFR was lower (0.47, 0.13, 0.28, respectively) than Pd/Pa and FFR (0.73, 0.23, 0.56 respectively). iFR was lower than Pd/Pa in all stenoses, by a mean of 0.08±0.07 units, p<0.001. Figure 2 shows the gain offered by iFR over Pd/Pa across the entire study.

### Postangioplasty stenosis evaluation

Coronary intervention was angiographically successful in all cases and physiological measures were only performed once angiographic or intracoronary imaging based optimisation had been performed. Mean residual stenosis after stenting, measured by QCA, was 14.1±8.2%. The mean of all three indices increased significantly after angioplasty (iFR 0.75±0.21 to 0.94±0.05, p<0.001; Pd/Pa 0.83±0.16 to 0.96±0.04, p<0.001; and FFR 0.66±0.14 to 0.89±0.07, p<0.001).

### Change in iFR, Pd/Pa and FFR after intervention

**Figure 3:**
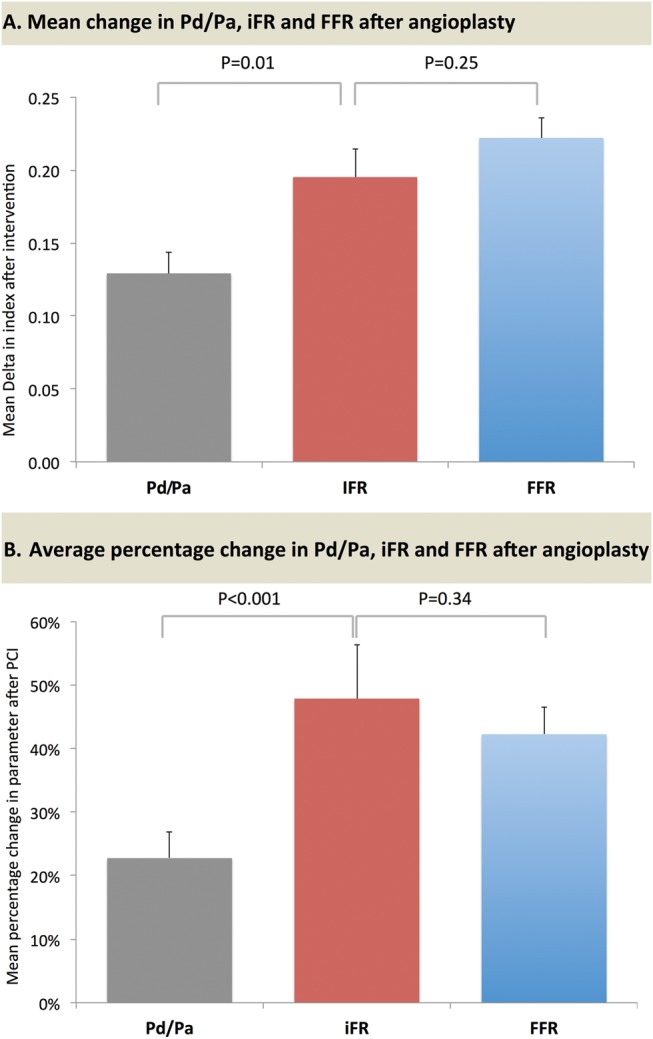
The mean change in preangioplasty and postangioplasty Pd/Pa, iFR and FFR values. Fractional flow reserve (FFR) and instantaneous wave-free ratio (iFR) and whole cycle Pd/Pa values before and after coronary angioplasty. Mean and standard error pre-PCI and post-PCI values are shown as vertical lines. Red horizontal lines represent stenoses which have a fall in index after PCI.

Across the whole study population, the change after intervention was significantly higher for iFR than Pd/Pa (ΔiFR 0.20±0.21 vs ΔPd/Pa 0.13±0.16, p=0.007) and was statistically similar for iFR and FFR (ΔiFR 0.20±0.21 vs ΔFFR 0.22±0.15, p=0.25, figure 3A). The magnitude of change elicited by PCI, that is the delta as a percentage of the pre-PCI value, was not significantly different between iFR (48±92%) and FFR (42±47%; p=0.34). The magnitude of change for Pd/Pa (23±45%) was significantly smaller than iFR (p<0.001) and FFR (p<0.001) (figure 3B).

The findings were unchanged when iFR and FFR agreed (iFR≤0.90, FFR≤0.80) in 103 stenoses, ΔiFR 0.22±0.21 vs ΔFFR 0.24±0.15, was not significantly different (p=0.57); both were significantly greater than seen with ΔPd/Pa (0.15±0.16, p<0.01 for both). In the 17 stenoses with iFR>0.90 pre-PCI, the ΔFFR was much smaller than when iFR≤0.90 (0.12±0.06 vs 0.24±0.15, p=0.002). Similarly, the ΔiFR was smaller (0.03±0.03 vs 0.22±0.21, p<0.001) but remained larger than seen with ΔPd/Pa (0.02±0.03, p<0.001).

After PCI, six stenoses had iFR values lower than FFR (with a difference of 0.05±0.04 in the value between the two indices). There was no difference in the QCA of these stenoses and the rest of the study population (9.8±2.1% vs 14.8±8.5%, p=0.24). In the subset of 26 stenoses in which pre-PCI iFR was lower than FFR, the change in iFR post-PCI was significantly greater than the change in FFR and Pd/Pa in the same lesions: ΔiFR 0.47±0.23 (magnitude 158±148%) versus ΔFFR 0.32±0.18 (magnitude 74±64%), p=0.01; versus ΔPd/Pa 0.33±0.18 (magnitude 70±76%), p=0.02.

### Stenoses remaining ischaemic or worsening after intervention

**Figure 4:**
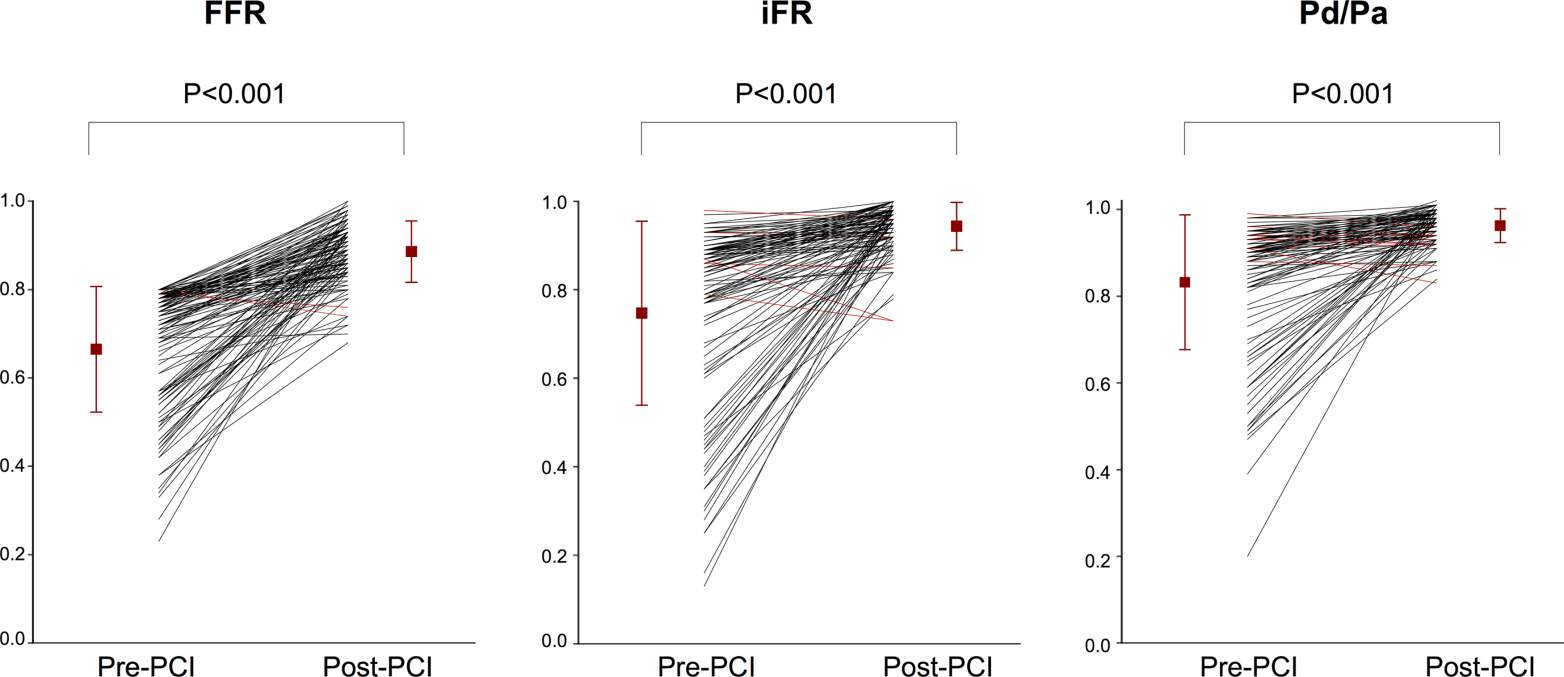
The change in preangioplasty and postangioplasty FFR and iFR values for individual stenoses. Fractional flow reserve (FFR) and instantaneous wave-free ratio (iFR) and whole cycle Pd/Pa values before and after coronary angioplasty. The red line shows the average pre and post values, while the error bars show SD.

The majority of lesions showed haemodynamic improvement after PCI. However, in a small number of cases the post-PCI values were lower. This was found in all indices in similar proportions (iFR:4%, FFR:2% and PdPa:7%, p>0.25 for all) (figure 4), with overall very small falls in index values (iFR:0.05±0.05, FFR:0.04±0.02, PdPa:0.02±0.02). Overall 15 stenoses had a FFR≤0.80 post-PCI (FFR 0.76±0.04) and 12 cases with iFR (iFR≤0.90; mean iFR 0.85±0.06).

### Lesion severity and impact upon physiological improvement

**Figure 5:**
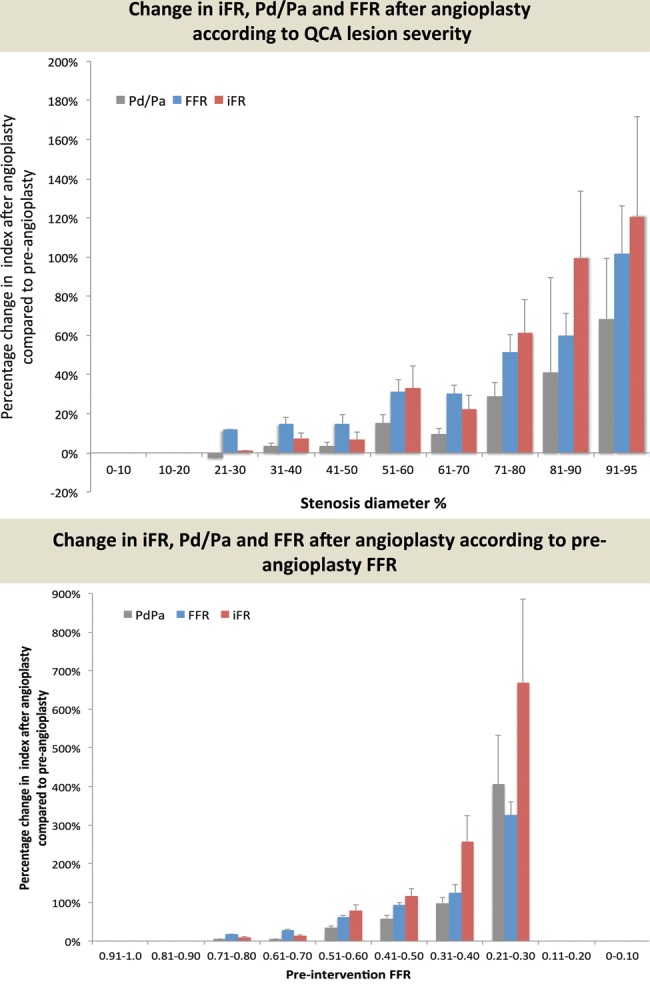
Improvement in iFR, Pd/Pa or FFR is closely associated with the angiographic severity of the stenosis. The change in index is shown as a percentage of the preangioplasty result (A) according to the lesion severity measured by percentage diameter stenosis and (B) by preintervention FFR value. A larger pre–post-PCI difference in iFR was observed in more severe lesions when compared with less severe lesions.

Pre-PCI lesion severity as determined by anatomical stenosis influenced the magnitude of physiological improvement (figure 5). Preangiographic QCA was related to the delta in physiological index (ΔFFR r=0.57, ΔiFR r=0.49, ΔPd/Pa r=0.51). Regression analysis demonstrated strongly significant positive relationship of initial lesion severity by QCA (percentage change in FFR R^2^ 0.26, iFR R^2^ 0.16, Pd/Pa R^2^ 0.16, each p<0.0001).

Post-PCI, residual stenosis severity measured by QCA had no strong relationship with either physiological measure (iFR r=0.24; FFR r=0.12). There was a modest relationship between the degree of improvement in angiographic severity (ΔQCA) and change in iFR (r=0.34) and change in FFR (r=0.40).

### Impact of diabetes, smoking status and hypertension on magnitude of increase of iFR and FFR post-PCI

No significant difference was observed when patients with hypertension, diabetes or smokers were compared with patients without these conditions (table 3). The change in iFR was significantly larger than Pd/Pa after PCI in patients with hypertension, those without diabetes and non-smokers.

**Table 3:** Patients with risk factors for microcirculatory disease have a similar change in iFR and FFR produced by PCI

		Delta pre–post-PCI				
Risk for microvascular disease	No of stenoses	FFR	iFR	Pd/Pa	iFR versus FFR	iFR versus Pd/Pa
Hypertension						
No hypertension	25	0.19±0.14	0.16±0.19	0.11±0.13	0.12	<0.001
Hypertension	95	0.23±0.15	0.20±0.21	0.13±0.16	0.06	<0.001
p Value		0.31	0.35	0.22		
Diabetes						
No diabetes	80	0.22±0.14	0.18±0.20	0.12±0.15	0.01	<0.001
Diabetes	40	0.23±0.17	0.23±0.23	0.15±0.18	0.71	<0.001
p Value		0.56	0.25	0.27		
Smoking status						
Non-smoker	72	0.16±0.19	0.18±0.19	0.12±0.15	0.11	<0.001
Smoker	48	0.26±0.15	0.22±0.22	0.15±0.16	0.09	<0.001
p Value		0.07	0.27	0.24		
Multiple risks						
No risk factors	9	0.14±0.13	0.13±0.17	0.09±0.12	0.85	0.04
Diabetic, hypertensive, smoker	13	0.22±0.14	0.22±−0.22	0.14±0.16	0.95	0.004
		0.22	0.37	0.38		

Delta in indices was compared according to the presence of hypertension, diabetes and smoking status. ANOVA was performed with post hoc testing and Bonferroni correction.

### Haemodynamic changes induced by PCI

The change in heart rate, measured during resting or hyperaemic conditions, after PCI had no relationship to the change in iFR (*R*^2^ 0.004) nor FFR (*R*^2^ 0.007). Similarly, change in mean arterial pressure after PCI had no relationship with change in iFR (*R*^2^ 0.004) nor FFR (*R*^2^ 0.002).

## Discussion

In this study, we found that in stenoses that typically undergo intervention (1) resting indices of stenosis severity can detect a change after PCI, with iFR and Pd/Pa values improving after successful PCI; (2) the change in iFR and FFR is similar; (3) the change seen is larger with FFR and iFR than seen with Pd/Pa.

Gruntzig's demonstration that resting trans-stenotic pressure gradients could detect change after angioplasty was limited by bulky low-fidelity pressure-sensing equipment.^10^ Hyperaemia improved sensitivity for whole-cycle averaged measures by increasing flow in stenoses which by definition must be non-flow limiting.^4^ iFR provides an alternative approach to increasing sensitivity, by automatically identifying a phase in diastole —*the wave-free period*—when trans-stenotic flow is highest during the resting cardiac cycle. Our findings confirm that using either of these approaches it is possible to observe a similar improvement in trans-stenotic pressure gradients after PCI.

### The challenges for pressure-only indices after PCI

Changes in trans-stenotic pressure gradients after coronary intervention, to remove an obstruction to flow, are a measure of the effect of the procedure.^10–12^ However, it is possible despite successful anatomical resolution of a coronary stenosis, unwanted or unaccounted for effects of PCI itself may pose difficulties for post-PCI physiological evaluation. Such effects included altered haemodynamics,^13^ changes in microcirculatory resistance,^14^ and altered responsiveness to adenosine due to microembolisation.^15^ The impact may vary according to initial lesion severity,^16^ ^17^ the stenting strategy^18^ and even concomitant drugs.^19^ Nonetheless, FFR has been shown to be of use to measure the incremental improvement in trans-stenotic gradient after balloon angioplasty, stent deployment, and following poststent high pressure balloon inflation and many of the theoretical concerns of a blunted microcirculatory response to adenosine after PCI have proved unfounded.^20–24^ The information has clinical utility also, with post-PCI FFR predicting restenosis and major adverse cardiovascular events.^20–22^ ^25–28^ While intravascular ultrasound (IVUS) and/or optical coherence tomography should remain the standard to assess quality of stent *deployment* and *apposition*, post-PCI physiology does reflect the end lumen area^29^ ^30^ and provides an assessment of the effects of the residual coronary disease to likely vessel ischaemia.

### Resting markers of lesion severity can detect lesion significance before PCI

Resting parameters are known to correlate with hyperaemic measures^31^ and experts agree that hyperaemia is not required for stenoses with significant resting gradients.^32^ By using only the diastolic wave-free period, iFR offers the lowest resistance over the cardiac cycle^1^ ^33^ and incremental benefit in the number of stenoses that can be safely assessed without adenosine.^34^ However, it was unclear whether resting measures would have sufficient dynamic range to detect improvement after PCI.

In this study, iFR as a resting index could distinguish improvement in stenosis severity similarly as FFR in a wide range of stenoses that would be selected for PCI. Pd/Pa also detected improvement albeit with a significantly smaller increment when compared with either FFR or iFR. All three measures, despite representing quite different physiological parameters, have a close correlation and therefore have clinical utility. However, as the onus falls upon the interventionist for demonstrating physiological functional gain after PCI, having a greater dynamic range, as offered by iFR and FFR, may be considered preferable. This is particularly pertinent to determine the significance of residual disease. The larger dynamic range offered by iFR and FFR over Pd/Pa means they have a greater range to diagnose stenoses and detect potentially small incremental improvements. If the potential dynamic range of improvement is small, important but smaller residual gradients may not be detected using a whole cycle Pd/Pa approach. Theoretically, this greater dynamic range may also be helpful in serial stenoses where greater discrimination is required to understand the impact of each stenosis and the effects of PCI to a given stenosis.

With FFR and iFR offering an equivalent magnitude of change on average for stenoses selected for intervention, procedures could be performed without vasodilators while being able to assess the change in an equivalent manner as when vasodilators are used. However, we did not seek to find an optimal ‘cut-off’ for post-PCI iFR that predicts outcome or vessel size. Further work with long-term follow-up and intravascular imaging is warranted.

In this study FFR<0.80 was used as the entry criteria. As a result, in some cases iFR and Pd/Pa were negative, while by study design FFR was always positive. In these cases, the rise in iFR and Pd/Pa was significantly smaller after PCI, than seen when iFR agreed with FFR. The implications as yet are unclear. Currently there is a significant body of evidence to support the FFR clinical cut-off value of 0.80. Further clinical studies are needed evaluate the clinical significance of these differences, and to assess the likely implications for clinical outcomes.

### Practical assessment of iFR after PCI

It is frequently thought that significant haemodynamic shifts caused by PCI may affect the ability of basal parameters to detect change after PCI. However, in this study, haemodynamic parameters at rest changed in a similar manner to those parameters measured during hyperaemia. Under resting and hyperaemic conditions, the absolute change after PCI was small and there was no relationship between the change in heart rate or blood pressure with the change in iFR or FFR post-PCI. This is similar to the relative heart rate and blood pressure independence reported in CLARIFY and by Johnson *et al*^4^ ^9^

Immediately after balloon deflation, dynamic changes associated with occlusive reactive hyperaemia may produce artificially lower iFR values. This is similar to injecting intracoronary nitrates or contrast.^11^ When this occurs it may lead to lower values of iFR. Reactive hyperaemia following transient balloon occlusion is similar to hyperaemia from intracoronary adenosine, typically lasting a short duration (5–30s) with a rapid return to basal conditions.^35–38^ Our finding show that provided post-PCI iFR measurements are made in a manner similar to FFR, that is after the deflated balloon is withdrawn and the guiding catheter is flushed, the hyperaemic effect is extremely short-lived and is of little clinical consequence as iFR and FFR have similar numbers of cases in which the post-PCI value is lower than the pre-PCI value.

### iFR values can be lower than FFR values in severe lesions

In this study sample of stenoses suitable for PCI, one in five (22%) stenoses had a pre-PCI iFR lower than FFR: that is the resting translesional pressure ratio was lower at rest than that achieved during stable maximal hyperaemia. While apparently counterintuitive, this phenomenon is well recognised in pressure-flow based studies, and occurs in all iFR-FFR comparator studies.^1^ ^2^ ^4^ ^8^ ^9^ The chance of an iFR measurement being lower than a FFR measurement increases with increasing disease severity. However, even in intermediate populations this occurs with a frequency of 8–15%. With increasing stenosis severity, where each patient undergoing PCI had a FFR <=0.80, the proportion of patients with a lower iFR than FFR increases significantly.

**Figure 6:**
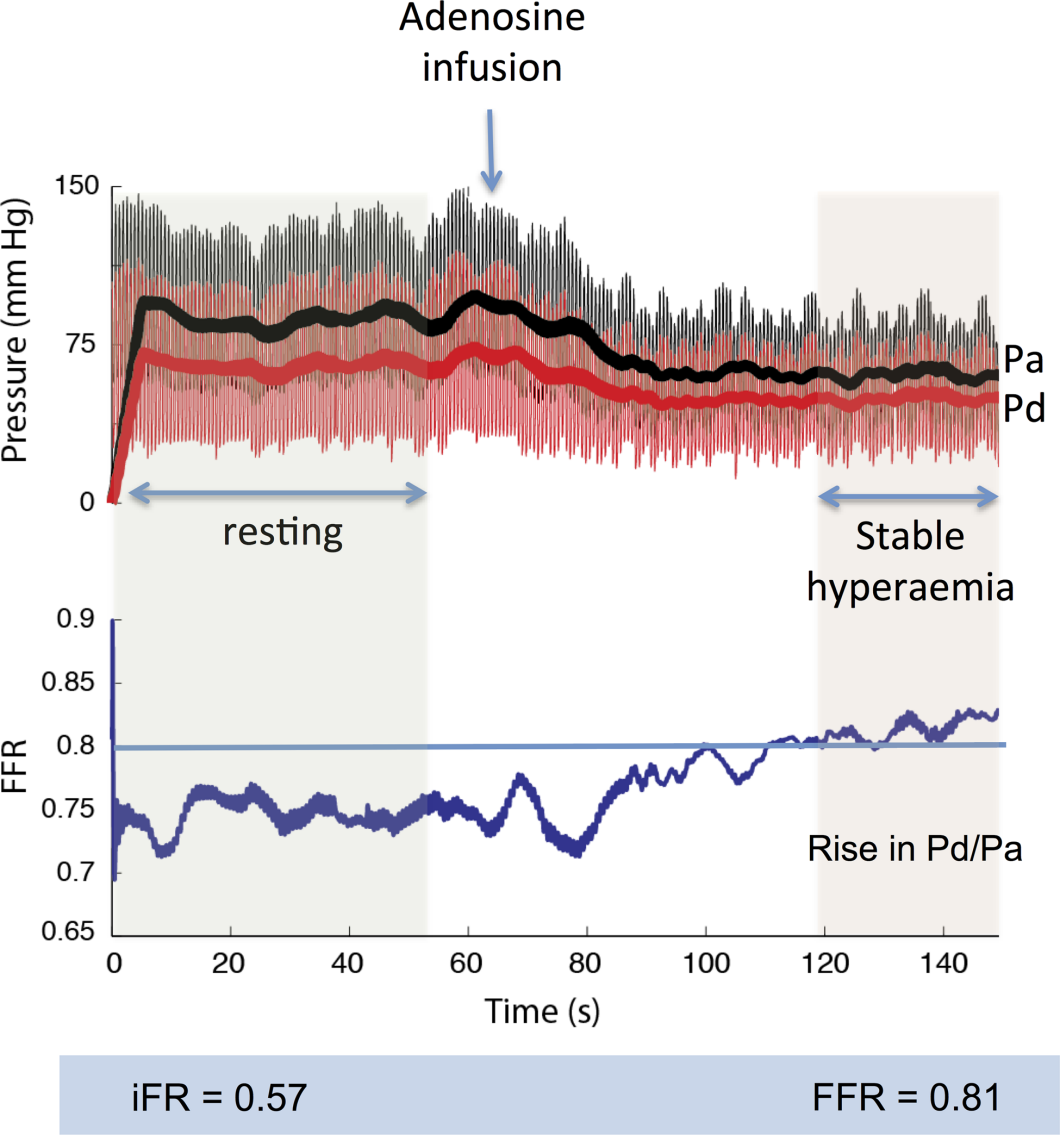
Resting gradients can be lower than hyperaemic gradients. The resting iFR is 0.57 and Pd/Pa is 0.75. Giving adenosine causes an apparent rise in the ratio, such that during stable hyperaemia, the FFR is 0.81. This likely represents adenosine mediated paradoxical vasoconstriction of the microvasculature.

However counterintuitive a lower resting than hyperaemic measurement may appear, the physiological principles underlying the phenomenon are well described.^17^ ^39^ ^40^ Coronary flow is maintained during resting conditions even in the presence of a coronary stenosis up to 90% because the microcirculation naturally vasodilates, reducing resistance, to preserve basal flow.^39–41^ In such severe stenoses, trans-stenotic flow is strongly influenced by proximal driving pressure which itself is maintained by autoregulation. In this setting, adenosine offers no additional vasodilation than naturally present, and resistance does not fall by as much as seen in non-flow limiting vessels.^4^ ^17^ However, the fall in central blood pressure particularly with intravenous adenosine can be sufficient to reduce the driving pressure across the stenosis with reduction in distal distending pressure. A loss in perfusion pressure leads to protective microcirculatory vasoconstriction, much like that seen in the peripheries during shock. This paradoxical vasoconstriction of the microvasculature leads to a rise in distal resistance^17^ ^42^ and may be sufficient to attenuate trans-stenotic gradients during stable hyperaemia. A typical case from the ADVISE study is demonstrated in figure 6 in which the resting iFR was 0.56 and the FFR 0.81.

The clinical implications of this paradox are unclear, particularly because the occurrence rate in trials such the FAME studies is unknown.^6^ ^7^ Furthermore, many cardiologists would choose not to give adenosine if the resting gradient is already below the treatment threshold, while some cardiologists choose to use lowest Pd/Pa ratio during adenosine infusion and thus may have inadvertently missed or disregarded rising FFR values caused by paradoxical effects of adenosine. Further assessment of this phenomenon is required and it is the subject of upcoming studies.

After PCI, an iFR lower than FFR could represent the effect of residual stenosis, but also of either residual hyperaemia as a consequence of balloon inflation, which may artificially reduce the iFR or the impact of microemboli preventing maximal hyperaemia in response to adenosine. Intracoronary flow velocity was not measured in this study, so it is not possible to identify whether an increase in flow (leading to a low iFR) or an increase in resistance (leading to a higher than expected FFR) was the likely cause of the differences between iFR and FFR. Overall this cohort was small, and there was no difference in the anatomical residual disease post-PCI to account for the six cases where iFR was lower than FFR.

### Relationship between anatomical stenosis severity and physiological pressure indices

It is intuitive that the potential for either iFR or FFR to increase following successful PCI was governed by the initial physiological severity of the lesion. In this study we also found angiographic significance demonstrated a relationship. This is likely to be due to our study design, where only patients with anatomical stenosis with FFR≤0.80 were included. If physiological assessment had been made pre–post-PCI in lesions deemed anatomically significant, with disregard for FFR, it is likely that the relationship between stenosis QCA and improvement in post-PCI physiology would have been significantly worse.

### Potential value and application of iFR to aid coronary angioplasty

Despite the utility of FFR, physiological guidance pre-PCI is performed in <10% of interventional cases.^43^ ^44^ Less data for post-PCI assessment is available but is likely to be a fraction of pre-PCI assessment.^45^ Unmet needs include the cost of pressure wires and reimbursement costs. Others are the time taken, the availability of vasodilators and certainty of reaching maximum hyperaemia.^46^ Even in the best hands, using intracoronary vasodilators in a single vessel adds a median of 9 (IQR 7–13) minutes onto a PCI procedure, while an infusion approach adds 11 (IQR 10–17) minutes.^47^ Multivessel assessment takes significantly longer.^47^ Simplification of measurements could promote physiological assessment in more vessels and in more patients. Documenting incremental changes in physiological markers may gain clinical importance as interventionists are increasingly required to document the appropriateness of the procedure^48^ and the benefit accrued.

### Limitations

FFR has been afforded a significant bias towards detecting a greater change because only patients with FFR≤0.80 were included. Therefore, while anatomically the lesions were moderate, many were severe physiologically as seen in the pioneering FFR trials.

FFR was measured using intracoronary adenosine in 20% of patients. This reflects the routine clinical practice and the interchangeable way in which adenosine is administered. While several studies have reported an overall excellent classification match between the techniques,^49^ it may have inadvertently introduced additional variability into the FFR measurements, which was not seen in the iFR arm which was always measured at rest prior to administration of adenosine.

Central venous pressure (CVP) correction of the simplified FFR calculation can improve the accuracy of the FFR measurement, though it is rarely performed clinically and in trials.^7^ ^25^ Future studies should consider formal assessment of the impact of CVP measurement upon the relationship between physiological indices, as well as defining the confidence boundaries of change by performing repeated measures before and after PCI.

Future work should also measure coronary wedge pressure to assess the impact of collateral vessels and the collateral flow index upon iFR measurement and how this affects iFR post-PCI. The differing effects of collateral vessels between resting and hyperaemic indices may explain important differences between iFR and FFR, and requires further assessment in future studies.

In keeping with routine clinical practice, this study did not measure coronary Doppler flow velocity, and therefore did not evaluate the specific impact of microcirculatory disease on flow-derived or pressure-flow derived indices.

### Conclusions

The incremental improvement in iFR following coronary angioplasty is similar to that of FFR and greater than Pd/Pa. Resting indices such as iFR have the potential to be used as objective measures of improvement in physiology following coronary angioplasty.
